# Evaluation of safety and efficacy of radiation-sterilized bone allografts in reconstructive oral surgery

**DOI:** 10.1007/s10561-012-9348-7

**Published:** 2012-12-13

**Authors:** Marta Krasny, Kornel Krasny, Artur Kamiński, Małgorzata Zadurska, Piotr Piekarczyk, Piotr Fiedor

**Affiliations:** 1 Department of Orthodontics, Warsaw Medical University, ul. Nowogrodzka 59, 02-006 Warsaw, Poland; 2 Medicare Dental Practice, Warsaw, Poland; 3 Department of Transplantology and Central Tissue Bank, Warsaw Medical University, Warsaw, Poland; 4 2nd Department of Cranio-Maxillofacial Surgery, Oral Cavity Surgery and Implantology, Warsaw Medical University, Warsaw, Poland; 5 Department of General and Transplantation Surgery, Transplantation Institute, Warsaw Medical University, Warsaw, Poland

**Keywords:** Alveolar reconstruction, Alveolar process atrophy, Preimplantation preparation, Bone grafting, Augmentation

## Abstract

Bone grafting allows reconstruction of the atrophied or destroyed alveolar process. In orthopaedics and traumatology allogeneic grafting has been used to restore defects of osseous tissue for over 60 years. In order to improve safety of the graft recipient, sterilized allogeneic grafts have been use. The aim of the study was to assess the direct and long-term outcomes following augmentation of atrophied alveolar processes with the use of radiation-sterilized allogeneic bone grafts. Sixty-eight patients were surgically treated between 2004 and 2011: 29 underwent open sinus floor elevation, post-extraction alveoli augmentation was performed in 16 subjects and 23 underwent reconstruction of the atrophied alveolar process. Augmentation of bone defects used bone granulate in 63 patients and bone blocks stabilized with titanium screws in 5 patients. PRF membranes collected from the patient’s blood were also used in all the procedures. In each of the cases optimal dimensions of the alveolar process were obtained allowing embedment of BIOMET 3I dental implant/-s. In all the patients the defects were successfully restored with implant-supported prostheses. Radiation-sterilized allogeneic bone grafts proved to be safe and effective for the patients and manageable for the surgeon constituting a good alternative to autogeneic material.

## Introduction

Lack of adequate mass of a patient’s own osseous tissue constitutes a clinical problem, still valid in dental implantology, which precludes intraosseous implant embedment. Bone grafting at the site of atrophied alveolar process is a first-line preparatory measure for implant-prosthetic treatment (Margonar et al. 2010).

Dental defects affect masticatory efficiency, deteriorate facial aesthetics and smile, and therefore cause psychological and physical problems resulting from the patient’s lowered self-esteem (Kiyak 2008; Taylor et al. 2009; Sierpinska et al. 2006). The stomach receives larger morsels, which leads to digestive discomforts (Langer 1976). Long-term absence of support zones results in disturbances within the line of occlusion through passive eruption of opposing teeth and consequently, temporomandibular joint complications (clicking, popping) as well as headaches of severity increasing over time (Hillier and Fam 1985).

Prosthetic treatment of an adult patient most commonly requires orthodontic correction aimed at improvement of occlusal conditions and positioning of the teeth in oral cavity. As we aspire to achieve the best possible aesthetic and functional outcomes, it is important to plan future permanent prosthetic restoration based on implants in an accurate and rational manner.

Procedures of atrophied alveolar process reconstruction before the implant-prosthetic treatment include open maxillary sinus floor elevation, increasing the width and/or the height of the alveolar process through augmentation of the post-extraction alveoli and filling of defects in the outer table of the compact bone formed following inflammatory conditions or destroyed during a traumatic extraction (Beck and Mealey 2010; Krasny et al. 2011).

Despite dynamic development of medical engineering, which provides a vast range of osteogenic and bone replacement formulations available on the medical market, a patient’s self-bone is still the safest and most durable material (Kao and Scott 2007; Rodella et al. 2011). However, some of the consulted patients do not agree to expand the area of the procedure beyond the range that is absolutely necessary, and doing so, they prevent the use of the procedure, which impels doctors to reach for allogeneic bone formulations. In allogeneic bone grafting, with the bone adequately prepared prior to the procedure, the atrophied or destroyed alveolar process may be expanded in width, height or filled. The procedure is considerably more delicate than the one using an autogenous bone as it reduces the operation sites to the recipient area only (the donor site is excluded). Duration of the procedure, pain discomfort, and intraoperative and postoperative burden are minimized and infections of the donor site are ruled out (Hardin 1994; Perrott et al. 1992).

Department of Transplantology and Central Tissue Bank of the Medical University of Warsaw have been preparing human tissue grafts for nearly 50 years. The grafts are prepared according to requirements described in the EU directives and national legal regulations in regard of tissue banking. The process of allograft preparation, is supposed to provide safety of their use as well as adequate quality of tissue, offering the assumed biological, physical, and biochemical properties (Dziedzic-Goclawska et al. 2008).

## Objective of the study

Assessment of immediate and long-term outcomes in augmentation of atrophied maxillary and mandibular alveolar processes with the use of allogeneic bone formulations.

## Materials and methods

**Fig. 1:**
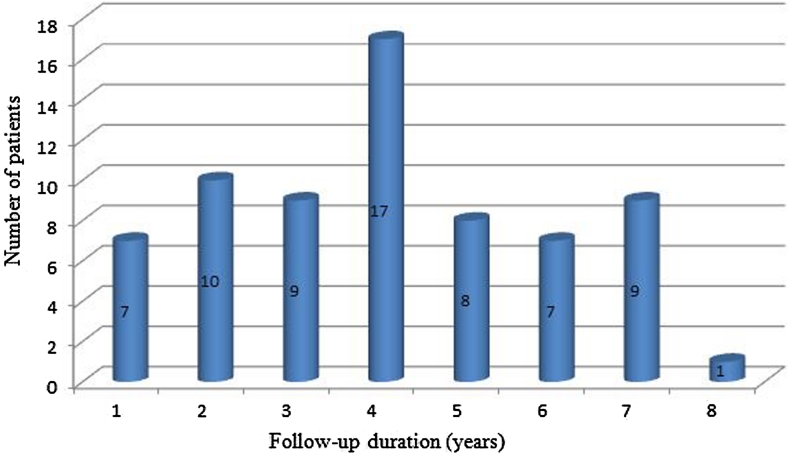
Follow-up periods for patients who underwent surgical procedure with the use of allogenic bone grafts

Reconstruction of the alveolar process prior to implant-prosthetic treatment with the use of allogeneic bone material was performed in 68 patients between 2004 and 2011. The group consisted of 28 females and 40 males aged 22–65. The follow-up period ranged from 1 to 8 years. On average efficacy of the grafting procedure was assessed 3 years after the procedure was done (Fig. 1). Three types of the procedures were performed with the use of allogeneic bone grafts: open sinus floor elevation, post-extraction alveoli augmentation and reconstruction of atrophied alveolar process.

The procedure of open sinus floor elevation with the use of allogeneic bone granulate was performed in group 1 in 29 patients qualified for that procedure based on the following criteria: minimal height of the alveolar process observed in the computed tomography (CT) scan: 4 mm, atrophy of the alveolar process (reduced height) from the side of the maxillary sinus and normal aeration of the maxillary sinus.

The procedure was performed under local anesthesia. The mucous membrane was incised at the top of the alveolar process and then the incision was extended to the vault of the oral vestibule. The mucoperiosteal flap was detached showing the anterior wall of the maxillary sinus above the site of planned implantation. With a diamond drill the bone lamella was removed creating an oval bone lid leaving the mucous membrane, which lines the sinus, untouched. The mucous membrane was subsequently dissected free and strengthened with PRF membrane, which significantly decreased the risk of perforation during augmentation (Tomford et al. 1990). Additionally PRF membranes were used in view of high concentration of osteogenic factors, which precipitated the graft remodeling process. The created bone cavity was filled with allogeneic bone granulate and the opening in the anterior sinus wall was also covered with the PRF membrane. The mucous membrane was then sutured.

In five patients embedment of the planned implants was performed simultaneously with augmentation, provided primary stabilization was obtained and the height of the self-bone exceeded 4 mm.

**Fig. 2:**
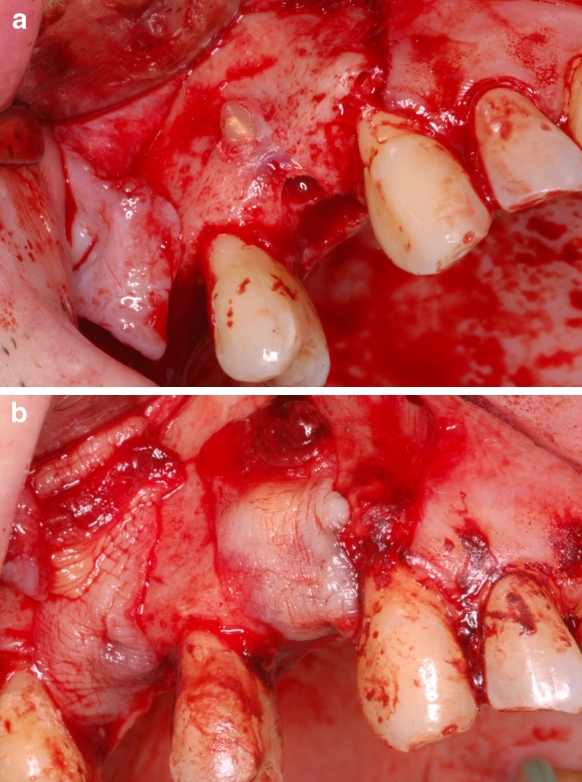
Patient M.Z. 39 years old: **a** perforation of the external lamina of alveolar process of maxillae, **b** augmentation of the bone defect with bone allograft covered with PRF membranes

The second group consisted of 16 patients, who underwent the procedure of post-extraction alveoli augmentation (Fig. 2a). Patients were qualified for this procedure based on the following criteria: X-ray and intraoral examination indicative of post-inflammatory defects of the outer table of the compact bone, X-ray indicative of considerable damage of the osseous tissue as a result of chronic inflammatory conditions and no signs of acute inflammation within the area of interest.

Augmentation with the bone granulate was performed following thorough curettage of the inflamed tissues and flushing the bone cavity with sodium chloride. After the material was applied, the entire area was covered with PRF membranes obtained from the patient’s own blood to accelerate formation of bone union and prevent epithelium from penetrating the area between the self-bone and the bone graft (Fig. 2b). The mucous membrane was mobilized in order to suture the wound without drainage.

In view of the persistent inflammation, bacterial infection of the site, and difficulties in obtaining primary stabilization, there were no cases of immediate implantation in group 2. Graft atrophy while forming a union with the patient’s bone could also expose a part of the implant, and doing so, deteriorate the subsequent aesthetic result.

**Fig. 3:**
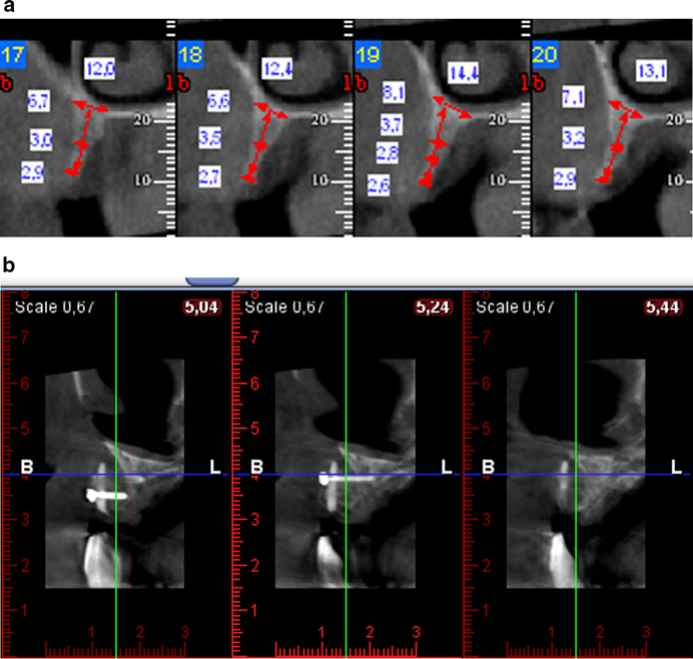
Patient A.B. 56 years old: **a** TK scans before grafting, **b** 6 months after grafting

Group 3 consisted of 23 patients diagnosed with atrophied outer table of the compact bone resulting from a traumatic extraction or inflammatory condition within the area. The subjects were enrolled for the procedure based on an intraoral examination as well as additional tests (X-ray, CT). The inclusion criteria were: minimal height of the alveolar process observed in the computed tomography (CT) scan: 8 mm (the length of the shortest implant), the width of the alveolar process below 5 mm and no signs of acute inflammation in the intraoral examination and additional tests (Fig. 3a).

**Fig. 4:**
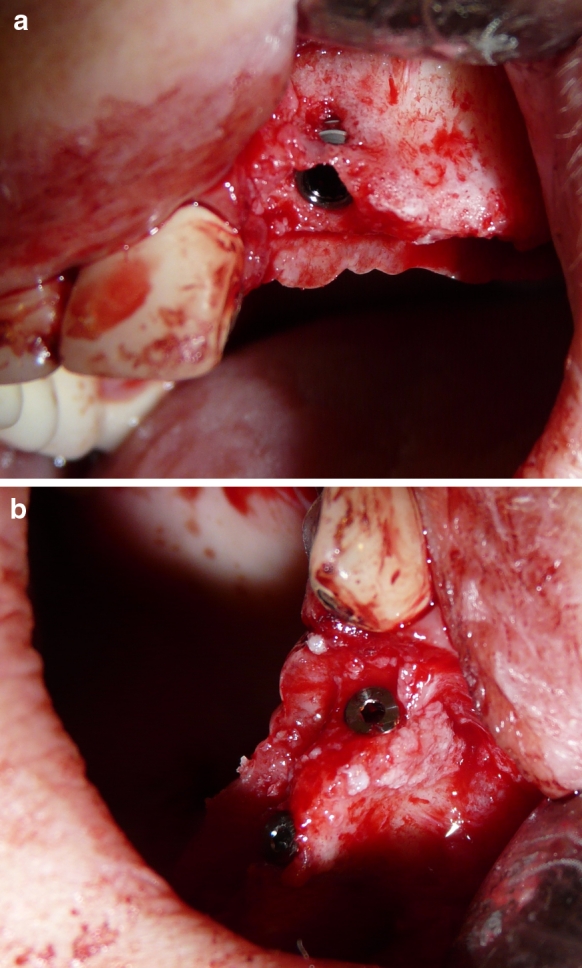
Patient K.R. 43 years old: **a** perforation of the external lamina of alveolar process of maxillae, **b** intraosseous implantation and simultaneous covering of the bone perforation with allogeneic granulate

The type of allogeneic bone formulation was selected based on the result of a CT scan assessing the degree of atrophy of the outer table. When the width of the process was slightly reduced below the minimum value—5 mm, with concurrent slight reduction in its height, augmentation was performed with the use of bone granulate covered with PRF membranes. In 11 out of 18 subjects the planned implants were embedded at the same time (Fig. 4a,b). In 5 cases, when an over 3 mm-defect of the outer table was found in its vertical dimension and/or over 4 mm-defect in its horizontal dimension, a bone block fixed with titanium screws and covered with PRF membranes was used to allow faster graft reorganization.

Demineralised bone matrix and allogeneic bone blocks were prepared in class C clean rooms in the Department of Transplantology and Central Tissue Bank. Both types of bone grafts were processed from bone tissue retrieved from deceased donors after they were screened (medical and social history, medical examination and autopsy results) and negative results of serologic testing were obtained. Frozen cortico-spongious bone blocks were prepared from the iliac ala. After being defatted in alcohol solution and rinsed, bone blocks were subsequently radiation-sterilized with a dose of 35 kGy. Whereas, the frozen demineralized bone matrix was prepared from the compact bone of diaphysis. After grounding and defatting, the bone grafts were decalcified in 0.6 M HCl and rinsed. The dose of 25 kGy was used for radiation-sterilization. For both graft types the radiation sterilization was performed with the accelerated electron beam in the Institute of Nuclear Chemistry and Technology in Warsaw (Dziedzic-Goclawska et al. 2005; Kaminski et al. 2010).

## Results

Clinical data obtained during long-term follow-up indicated that allograft surgical procedures were equivalent or superior to autograft transplantation (Shafiei et al. 2009).

All the patients underwent augmentation performed by the same operator and procedures. No peri-operative complications were reported in any of the study groups.

The first follow-up examination was held after 12–14 days of the procedure. The following parameters were assessed: the color of the mucous membrane within the area of interest, the degree of healing in tissues, occurrence/lack of wound drainage and/or edema, patient-reported pain discomforts. On the same day the sutures anchoring the wound edges were removed.

In case of any discomforts within the operational site the patients attended another follow-up visit after 3 months. This time is required for remodeling and integration of the graft with self-tissue. Based on the intraoral assessment and follow-up X-ray normal bone union was found in all the patients. At the same time a procedure of the planned implant embedment was performed allowing also intraoperative confirmation of diagnosis.

The third follow-up visit after the reconstruction of the alveolar process depended on its location. In case of mandibular location it was held following 3 months of the implant embedment during the stage of prosthetic restoration of the dental defect, whereas, in case of maxillary location the time of implant integration with the bone is longer—the process lasts for 6 months; therefore graft follow-up was held after 6 months of implant embedment in the bone, also during the stage of prosthetic restoration of the dental defect.

In case of reconstruction of the atrophied alveolar process through sinus floor elevation with simultaneous implantation, the efficacy of the procedure was assessed after 2 weeks of the follow-up visit combined with removal of the sutures, after 1 month of the first follow-up and after the minimum of 9 months at the time, when the second prosthetic stage of the implant-based restoration was performed.

The use of a bone block in group 3 disqualified these patients from the simultaneous procedure of implantation and in such cases the follow-up visits were held according to the outline presented above. The other group 3 subjects attended follow-up visits after 2 weeks, after 6 weeks of the procedure and after 3 months during implant embedment, and subsequently during the prosthetic stage of the work after 3 or 6 months depending on the implant location.

In all of the subjects adequate height and width of the alveolar process was obtained (Fig. 2b), allowing embedment of BIOMET 3I implant/-s and in all the cases the implant-prosthetic restoration of the defect was successful.

During follow up period, presented in Fig. 1, no cases of pathological atrophy of the bone or gums recession were observed. Neither mobility, nor dental implant loss were found. Long-term observation presented in the diagram confirms the efficacy of the presented procedure in case of a narrow alveolar process.

## Discussion

Both, the clinical experience of the authors and literature, imply that, although the autogenous material is the safest and biocompatible, allogeneic formulations, in view of their availability, indefinite amount and ease of application, are used in cases of extensive or numerous sites of bone tissue atrophy in processes (Margonar et al. 2010). Comparatively large amount of bone tissue may be obtained from the iliac ala but the procedure creates burden for the patient and must be performed under general anesthesia. The urge to minimize complications and adverse effects of the general anesthesia forces the operator to use intraoral donor sites, i.e. the area of the mentum or retromolar triangle, which provide limited amount of the material, hence reducing indications for such procedure (Chaushu et al. 2010).

Owing to reduction of the operational sites (elimination of graft harvesting procedure) the number of complications related to augmentation with the use of allogeneic bone was reduced considerably. The most common ones associated with self-tissue harvest included transient mandibular nerve paralysis, problems related to wound healing and/or wound breakdown. Due to considerably poorer patient’s hygiene inflammation of the mucous membrane within the operational site was commonly observed, which hindered and extended the recovery time (Krasny et al. 2011). A serious complication of the procedure may result from inaccurate assessment of individual anatomy of the mandible, possibly leading to exposing or damaging of the tooth apices within the donor site.

Allografts have been widely used in orthopedic surgery for a long time for many clinical applications including tumors, revision arthroplasty, trauma, spine fusion and nonunion (Komender et al. 1996; Albert et al. 2006). The use of autografts in orthopedic surgery is not frequent nowadays. Bone allografts provide a safe and efficient alternative (Albert et al. 2006). The use of allogeneic biostatic tissue grafts is beneficial; however, it may involve some risk, e.g. due to possible transmission of infectious diseases (Tomford et al. 1990; Simonds et al. 1992). No symptoms of infectious disease transmission were found in patients described in this study. To assure the safety of application of human tissue grafts irradiation was introduced in Poland in 1963 as a routine procedure for sterilization (Dziedzic-Goclawska et al. 2005). No deleterious effect of radiation-sterilization on physical and biological properties of tissue allografts has been confirmed in laboratory and clinical studies (Dziedzic-Goclawska et al. 2005; Komender et al. 2001).

Various materials for guided bone regeneration, alveolar processes regeneration in particular, are commonly used worldwide. The materials include autologous, allogeneic and xenogeneic products as well as artificial materials (Cicciu et al. 2012; Nissan et al. 2011; Worth et al. 2005; Cordaro et al. 2008; Perrott et al. 1992). The recent years have brought introduction of advance therapy medicinal products, which, apart from the scaffold, contain autologous osteogenic cells. In some countries the products used, not yet registered in Europe, contain active substances, cytokines, in addition to the scaffold. Our study evaluated radiation-sterilized human allografts (DBM and bone blocks) for efficacy. Some authors believe that radiation-sterilized grafts show reduced mechanical durability and greater tendency to resorption compared to those, which were not sterilized (Eastlund 2005; 2006). Our observation unquestionably confirms that radiation-sterilized grafts may be used for reconstruction of atrophied alveolar processes, where mastication forces may reach 80 kg at the small area of the alveolar process.

Remodeling of the inserted radiation-sterilized bone graft was assessed exclusively on the basis of radiological imaging and macroscopic examination during implant embedment. No biopsy of the recipient site was performed and therefore no histological specimens were processed, which, when analyzed, might have constitute supplementary material for clinical observations. The authors will consider histological and morphometric assessment of graft remodeling when planning further studies on radiation-sterilized allogeneic bone grafts.

Demineralized, irradiated bone, which does not tolerate axial loading, was used for sinus-lift and post-extraction alveoli augmentation procedures at low-stress areas (Krasny et al. 2011; Kao and Scott 2007; Hardin 1994). Allogeneic irradiated frozen bone blocks, that possess similar properties as autologous ones, were used for reconstruction of alveolar processes. Preliminary results of application of radiation-sterilized bone blocks are similar to those, where fresh frozen allografts were used (Contar et al. 2009).

Owing to the PRF (Plasma Rich Fibrin) membrane, which contains large number of growth factors, the process of graft union with recipient’s own bone was accelerated. Due to elasticity and viscosity these membranes adhere to the bone surface acting as mechanical barrier against the penetration of the endothelium (Simon et al. 2009).

Presented results strongly suggest that radiation-sterilized bone allografts can be successfully used for reconstruction in oral surgery procedures. Allogeneic bone material is a good bone replacement formulation in case of maxillary and mandibular alveolar process reconstruction and may be used in out-patient healthcare. The use of allogeneic grafting reduces the number of operational sites and minimizes the physical burden for the patient. The use of PRF membranes allows fixation of bone allografts as well as faster union with the patient’s self-bone. Orthodontic treatment facilitates scheduling and performance of augmentation and allows ultimate implant-prosthetic restoration of the dental defect improving the final aesthetic outcome.

## Conclusion

The ease of application and availability of the material allows grafting within a considerable area of the facial skeleton. Long-term clinical outcomes confirm the safety of this surgical procedure allowing permanent restoration of dental defects.

Our data confirm that surgical procedure with radiation-sterilized bone allografts constitutes a very effective and promising treatment modality in patients with alveolar atrophy.
